# Impaired Reversal Learning in APPPS1-21 Mice in the Touchscreen Visual Discrimination Task

**DOI:** 10.3389/fnbeh.2019.00092

**Published:** 2019-05-09

**Authors:** Lore Van den Broeck, Pierre Hansquine, Zsuzsanna Callaerts-Vegh, Rudi D’Hooge

**Affiliations:** Laboratory of Biological Psychology, KU Leuven, Leuven, Belgium

**Keywords:** Alzheimer’s disease, mouse models, touchscreen operant cages, reversal learning, behavioral phenotyping

## Abstract

Preclinical-clinical translation of cognitive functions has been difficult in Alzheimer’s disease (AD) research but is crucial to the (predictive) validity of AD animal models. Reversal learning, a representation of flexibility and adaptability to a changing environment, might represent such a translatable feature of human cognition. We, therefore, examined visual discrimination (VD) and reversal learning in the APPPS1-21 mouse model of amyloid-based AD pathology. We used touchscreen operant cages in novel and translationally valid, as well as objective testing methodology that minimizes within- or between-trial handling. Mice were trained to associate a visual cue with a food reward (VD learning), and subsequently learned to adjust their response when this rule changed (reversal learning). We assessed performance at two different ages, namely at 6 months of age, considered an early disease stage, and at 9 months, a stage of established pathology. Both at 6 and 9 months, transgenic animals needed more sessions to reach criterion performance, compared to wild-type controls. Overall, transgenic animals do not show a general cognitive, motivational or motor deficit, but experience specific difficulties to adapt to reward contingency changes, already at an early pathology stage.

## Introduction

Translatability of testing procedures that enable the examination of clinical symptoms across the preclinical-clinical spectrum, remains a concern in many areas of contemporary neuroscience and pharmaceutical development. Many currently used animal procedures are fundamentally different from the methods used in human neuropsychology, which could be a reason why preclinical findings often fail to translate to the clinical condition (Bussey et al., 2012; McGonigle and Ruggeri, 2014; Nithianantharajah et al., 2015). Cognitive abilities have been difficult to compare between species, but an organism’s ability to adapt its behavioral repertoire to changing contexts and situations could be such a translatable feature of cognition (Auersperg et al., 2012). Behavioral flexibility is based on higher cognitive abilities (van Schaik and Burkart, 2011), which can be examined experimentally using reversal learning tasks that require test subjects to respond appropriately and adaptively to reversed reward contingencies (Emery and Clayton, 2004; Roth and Dicke, 2005; Auersperg et al., 2012; Klanker et al., 2013). In addition to their translational validity, modern-day preclinical procedures also need to be welfare friendly (e.g., low stress) and objective (e.g., relatively free from bias and automated, Bussey et al., 2012).

The translational gap has proven to be especially pertinent in Alzheimer’s disease (AD) research (Cummings et al., 2014; Reiman, 2017). During the last 15 years, 99.6% of all clinical trials in AD drug development failed, which is at the lowest end of all therapeutic areas (Cummings et al., 2014; Hay et al., 2014). These failures have been attributed, at least partly, to the limited predictive validity of preclinical results, which explains the continued interest in translatable AD models and test procedures (Romberg et al., 2013; Windisch, 2014). Incidentally, executive dysfunction and concomitant defects in cognitive flexibility are affected in mild cognitive impairment (MCI), the precursor to AD (Amieva et al., 2003; Ready et al., 2003; Tranter and Koutstaal, 2008; McGuinness et al., 2009). These defects become even more prominent and pathognomonic as MCI develops into full-blown AD (Tabert et al., 2006; Crowell et al., 2010).

Behavioral or cognitive flexibility has scarcely been studied in preclinical AD models. We, therefore, used touchscreen operant cages to examine visual discrimination (VD) and reversal learning abilities in the APPPS1-21 mouse model of amyloid-based AD pathology (Radde et al., 2006). Operant cages fitted with touchscreen devices allow animal researchers to assess complex cognitive abilities, such as discrimination and reversal learning, similar to human computer-based assessment procedures (e.g., Cambridge Neuropsychological Test Automated Battery, CANTAB). Since reversal learning ability has been proposed as a predictive, early marker in clinical AD (Binetti et al., 1996; Chudasama, 2011; Van Harten et al., 2013), we assessed VD and reversal learning in APPPS1-21 mice at two different ages. We and others have shown that amyloid-β plaque deposition starts in the frontal cortex of these mice at the age of 2 months, extending to hippocampus at around 4 months, eventually affecting all brain regions (except the cerebellum) by the age of 7–8 months (neuropathology is said to be full-blown at this stage). However, the first signs of cognitive impairment have only been described at the stage of full-blown neuropathology (Radde et al., 2006; Serneels et al., 2009; Lo et al., 2013). Previous testing revealed impairments in spatial learning and memory, as well as defects in passive avoidance learning and social memory (Serneels et al., 2009; Lo et al., 2013). In the present report, we tested APPPS1-21 mice at 6 months of age, which can be considered an early stage in terms of behavioral symptomatology and neuropathology, and at 9 months, a more advanced stage. Since cognitive flexibility is affected early on in human AD patients, we expected our transgenic animals to show reduced reversal performance already at the earliest stage.

## Materials and Methods

### Animals

We bred two batches of mice on a C57BL/6J background, comprising APPPS1-21 transgenic mice and age- and gender-matched wild-type littermates, tested at 6 and 9 months of age. Heterozygous APPPS1-21 mice co-express the Swedish mutation K670M/N671L of the amyloid precursor protein (APP) and the mutated human presenilin-1 (PS-1) L166P (Radde et al., 2006). We chose not to test our mice longitudinally because the extent of the learning defect would have been more difficult to interpret in a longitudinal experiment due to carry-over effects. Mice can retain certain procedural aspects of a task for quite a long time, repeated testing would therefore not start from the same naïve level. Also, repetitive reversal learning would enhance overall learning speed, since mice are known to acquire an aspect of the meta-procedural knowledge that carries over to repetitive reversal sessions. It would have been unclear how repetitive performance interacted with the progressive nature of the pathology. Moreover, repetitive testing would generate a condition of cognitive enrichment, which has been shown to influence test performance. The group tested at 6 months consisted of 23 wild type (13 females, 10 males) and 19 APPPS1-21 mice (10 females, nine males), whereas the group tested at 9 months consisted of 19 wild types (10 female, nine males) and 20 APPPS1-21 mice (10 females, 10 males). One animal died during experimentation (APPPS1-21 mouse of 9 months of age), and was excluded from the analysis. Genetic status of the mice was confirmed by PCR genotyping on isolated DNA from ear biopsies (Radde et al., 2006). All animals were group-housed in standard animal cages and under conventional laboratory conditions (12 h light/dark cycle, lights on at 8 a.m., 22 ± 2°C and 40%–50% humidity). The experiments were conducted during the light phase of their circadian cycle and efforts were made to test, weigh and feed the animals at the same time each day. Behavioral assessment started 10 days before their designated age condition, and 48-session cut-off was implemented because of age constraints. All protocols have been approved by the animal ethics committee of the University of Leuven, Belgium, in keeping with European directives. Throughout the testing period, mice were kept at 85%–90% of their free-feeding weight by scheduled feeding (which was maintained by 1 h of feeding per day) to ensure sufficient motivation during touchscreen testing.

### Apparatus and Procedure

Animals were tested in eight operant cages (Campden Instruments Ltd., Leics, UK), equipped with a touchscreen device (touch was registered by infrared photocells) displaying predefined visual cues controlled by ABET II Software (Lafayette, IN, USA). The chambers were equipped with infrared beams in front of the screens that displayed the stimuli. Thus, touching the screen without any pressure was enough to register their response (subjects often did touch the screen with their nose). Further behaviors inside the chambers were recorded by an internal IR camera allowing live monitoring of the mouse. Furthermore, the setup was equipped with a liquid food dispenser, a house light and sound generator. The cages were placed inside ventilated sound and light attenuating boxes. All animals were eventually subjected to two consecutive protocols as previously described (Bussey et al., 2012; Mar et al., 2013; Romberg et al., 2013; Horner et al., 2013): (i) learning to associate correctly a visual cue with a food reward (VD); and (ii) learning to adjust response when the rules changed (i.e., reversal learning). All daily sessions lasted either 30 trials or maximally 60 min. At the end of each testing day, the operant chambers (liquid food dispensers and tubes, plastic walls, grid floors and waste trays) were thoroughly cleaned and rinsed with warm water and 70% ethanol.

Training started with a habituation phase to get the animals acquainted with the test conditions and environment, followed by a pre-training phase to teach the animal to initiate a trial and to make correct instrumental responses. After reaching the criteria of correctly initiating a trial and responding on three successive trials, VD training was started, during which animals had to discriminate between a correct (CS+) and an incorrect (CS−) stimulus to obtain a reward (i.e., a drop of strawberry milk). The stimulus location (left or right on the screen) was pseudorandomized such that the same stimulus was never shown more than three times in a row at the same location. Incorrect responses to this regular trial (RT) resulted in a correction trial (CT; i.e., stimulus is presented in the same location until the subject responds correctly). CTs did not count towards the trial limit or the percentage accuracy. When the animals reached the pre-defined performance criterion (i.e., performing 30 trials at 80% correct for two consecutive days), the final reversal phase was initiated, during which the reward contingencies of CS+ and CS− were switched (REV). Notably, an individual criterion-based protocol was adopted, indicating that each animal transitions to the next phase at its own rate. The goal was to train each animal to a high and steady performance level during VD before initiating reversal training to be able to compare reversal performance. The reversal phase was identical to the VD phase but with reversed reward contingency, forcing the animal to adjust its learned responses.

### Statistical Analysis

*T*-tests for independent samples were used to assess general performance on shaping, VD and reversal learning (REV). Therefore, the total number of sessions to criterion were compared between wildtype and transgene animals. Mice that did not reach criterion in a specific stage, were not included in the respective analysis. Repeated measures analysis of variance (rmANOVA) assessed the significance of differences in performance over consecutive days (number of trials, percentage accuracy, and number of CTs) with Bonferroni *post hoc* test for pairwise comparisons. As a consequence of allowing individual trajectories, however, missing values inevitably occurred. Because rmANOVA cannot handle missing data, we included dummy values that were calculated as the average of the last two sessions of the respective stage. We realize that the use of dummy variables is contentious, but this procedure was included to circumvent problems of missing values. The major assumptions are, of course, that performance would have been stable during continued testing, and that a period of inactivity would not result in changes in response rates. Both assumptions seem to be fairly robust (Mar et al., 2013). We could have used a group criterion to proceed to the next phase, which would have meant that, before proceeding to the primary task phase or probe tests, acquisition-phase training would continue until all animals reached criterion. However, a major disadvantage of this approach would have been that some animals would be overtrained and have obtained more rewards. Therefore, we chose to use an individual criterion to avoid carry-over effects (i.e., move each animal into the primary task phase or probe tests immediately after it has reached the acquisition phase criterion). Furthermore, rmANOVA is not robust against violations of the sphericity assumption. Therefore when the assumption of sphericity was violated, the Greenhouse-Geisser correction of the degrees of freedom was applied. The criterion for statistical significance was *p* < 0.05.

## Results

**Figure 1:**
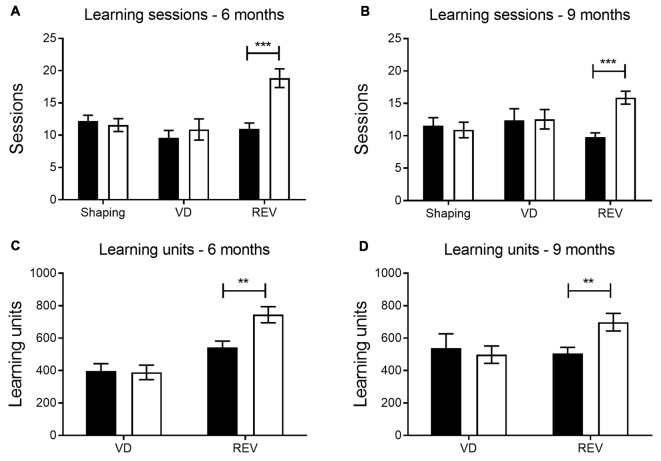
General performance in touchscreen protocol by phase. Number of sessions at 6 **(A)** and 9 months **(B)** during the shaping and the visual discrimination (VD) phase were similar in APPPS1-21 (white bars) and wild-type controls (black bars). However, during reversal learning (REV) APPPS1-21 mice performed worse, indicated by a significantly higher number of sessions required to reach criterion performance. During VD, the total number of learning units (RT + CT) needed to reach criterion performance did not differ between genotypes at 6 **(C)** and 9 **(D)** months. However, during reversal learning, APPPS1-21 mice at both ages needed significantly more learning units to reach criterion. Data plotted as mean ± standard error of mean (SEM), *p < 0.05, **p < 0.01, ***p < 0.001 (Bonferroni *post hoc* test).

The number of sessions needed to reach performance criterion was used as a general measure of learning (i.e., more sessions before criterion indicated more difficulty in learning the reward contingencies associated with the stimuli). When comparing the number of sessions to criterion between transgenic and wild-type animals, transgenic APPPS1-21 mice at 6 and 9 months of age needed more sessions during reversal learning (REV, Figures 1A,B, *p* < 0.001 and *p* < 0.001, respectively). In contrast, their performance during shaping and VD was similar to wild-type controls (shaping and VD phase at 6 months, *p* = 0.63 and *p* = 0.51, and at 9 months, *p* = 0.69 and *p* = 0.95, respectively).

At 6 months the total number of learning units (RT + CT trials across VD sessions), needed to reach criterion, did not differ significantly between transgenic and wild-type mice (*p* = 0.90; Figure 1C). Also at 9 months, we do not find a significant difference between conditions in VD (*p* = 0.68; Figure 1D). For reversal learning, however, transgenic animals require significantly more trials in total to reach criterion, both at 6 and at 9 months (*p* = 0.005 and *p* = 0.008, see Figures 1C,D, respectively).

**Figure 2:**
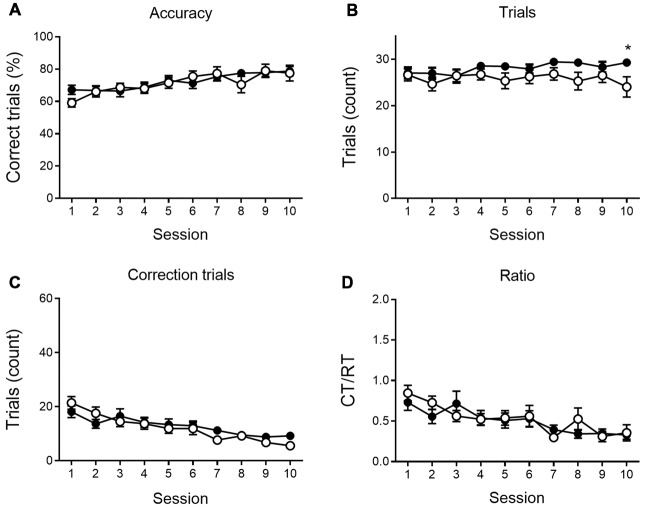
VD performance at 6 months of age. APPPS1-21 mice (white symbols) show learning performance similar to wild-type controls (black symbols) without motivational decline. No main effects of genotype were found for accuracy of performance (**A**; shown as the percentage correct responses), the number of trials performed per session **(B)**, the number of correction trials (CTs; **C**) and the CT/RT ratio **(D)**. Data plotted as mean ± SEM, *p < 0.05 (Bonferroni *post hoc* test, APPPS1-21 vs. wild type controls).

**Figure 3:**
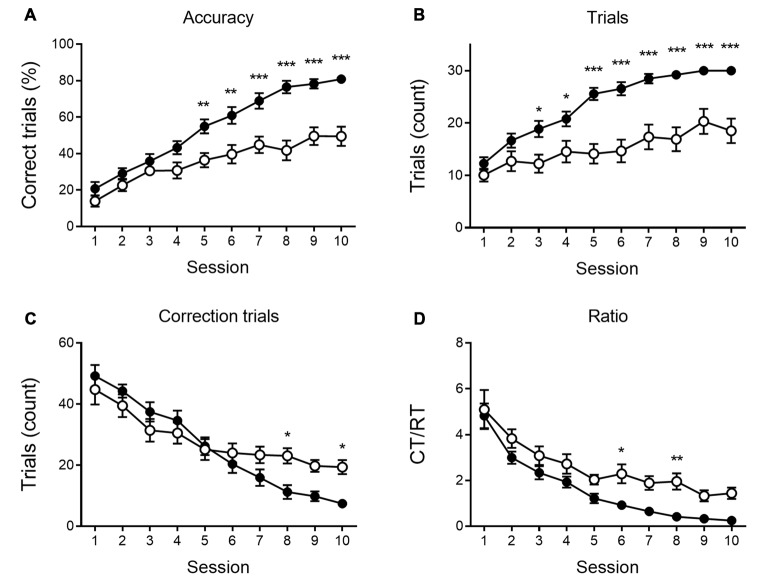
Reversal learning performance at 6 months of age. Reversal learning deficit in APPPS1-21 (white symbols) compared to wild type controls (black symbols). While both genotypes showed a steep increase in accuracy of performance over sessions **(A)**, APPPPS1–21 were less successful. A difference in performance became apparent after 4 test days when APPPS1-21 mice started lagging behind (significant interaction between sessions and genotype, see text for details). The number of regular trials (RTs) per session showed a significant increase over sessions **(B)**. Starting from session 3, however, transgenic animals perform fewer RTs. The number of CTs decreases significantly over sessions as animals learn the reversed reward contingency **(C)**. Starting from reversal day 7, transgenic animals perform significantly more CTs, indicating their greater difficulty abandoning the previously learned reward contingency. The CT/RT ratio decreased significantly over sessions in both groups **(D)**. Data plotted as mean ± SEM, *p < 0.05, **p < 0.01, ***p < 0.001 (Bonferroni *post hoc* test, APPPS1-21 vs. wild type controls).

We further analyzed the number of RTs, the accuracy of responding and the number of CTs during the VD (Figure 2) and reversal phase (Figure 3), and the CT/RT ratio across consecutive sessions. Responses are already slightly about chance level during the first session due to intra-session learning. Mice learn this VD task fairly quickly, and learning already takes place during the first session. As illustrated in Figure 2, 6-month-old animals show a significant learning effect (main effect of time) during VD, as the percentage correct responding increases (*F*_(9,360)_ = 7.69, *p* < 0.001), and the number of CTs (*F*_(9,360)_ = 13.50, *p* < 0.001) and the CT/RT ratio (*F*_(9,360)_ = 8.00, *p* < 0.001) decreases across sessions.

None of the indicated measures show a main effect of genotype during VD, indicating that APPPS1-21 mice are able to learn and perform the task properly and do not show a motivational decline. When looking at the reversal learning phase, a distinct deficit in the APPPS1-21 animals is present (Figure 3). We find a clear learning effect in all measures (main effects of session for percentage correct: *F*_(9,342)_ = 55.27, *p* < 0.001; number of RTs performed: *F*_(9,342)_ = 37.97, *p* < 0.001; number of CTs performed: *F*_(9,342)_ = 44.70, *p* < 0.001 and CT/RT ratio: *F*_(9,342)_ = 38.85, *p* < 0.001), we find a significant genotype effect in all measures as well (except number of CTs), with transgenic animals consistently performing worse than their wild-type controls (main effects of genotype on percentage correct: *F*_(1,342)_ = 27.38, *p* < 0.001; number of RTs performed: *F*_(1,342)_ = 27.78, *p* < 0.001 and Ct/RT ratio: *F*_(1,342)_ = 17.07, *p* < 0.001). Also interaction effects can be seen in Figure 3, transgenic and control mice increase similarly in performance during the first 4–5 sessions, after which the controls start to outperform the transgenic animals (accuracy × genotype: *F*_(9,342)_ = 5.91, *p* < 0.001; interaction trials × genotype: *F*_(9,342)_ = 5.96, *p* < 0.001, and interaction CTs × genotype: *F*_(9,342)_ = 4.49, *p* = 0.037). Only for Ct/RT ratio, this interaction is not significant (interaction ratio × genotype *F*_(9,342)_ = 0.76, *p* = 0.65). However, as shown in Figure 3D, this same trend is apparent in the ratio data.

**Figure 4:**
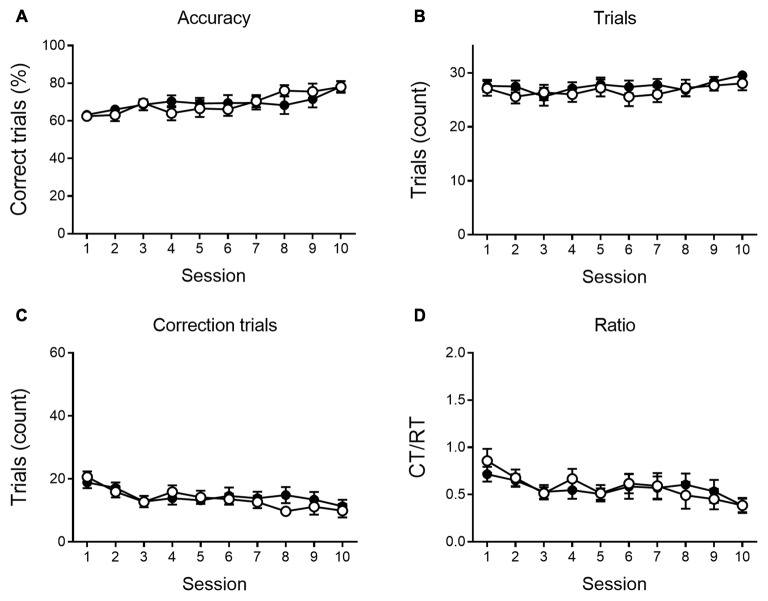
VD performance at 9 months of age. At 9 months of age, APPPS1-21 mice (white symbols) are able to learn the VD task similar to wild type controls (black symbols). Percentage of correct responses increased significantly for transgenic and control animals **(A)**. The number of trials performed per session does not show any main effect of session nor genotype **(B)**. The number of CTs performed **(C)** and the CT/RT ratio **(D)** across sessions diminished at a similar rate in both genotypes. Data plotted as mean ± SEM.

**Figure 5:**
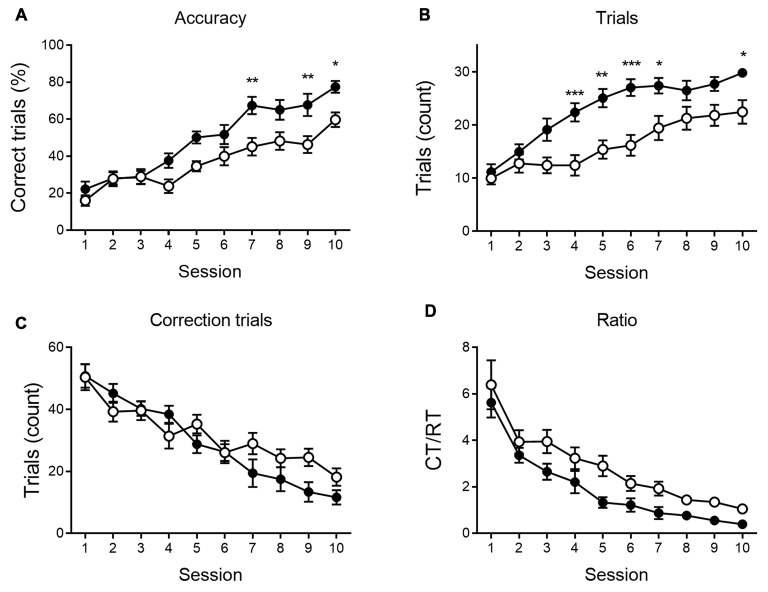
Reversal learning performance at 9 months of age. During reversal learning, APPPS1-21 (white symbols) learned slower **(A)** and needed more trials **(B)** than wild type controls (black symbols). Wild type animals outperform APPPS1-21 after 3–4 sessions. While the number of CTs decreases significantly over sessions, APPPS1-21 need more CTs (significant interaction effect between days elapsed and genotype) as they have more difficulty abandoning the previous reward contingency **(C)**. While the CT/RT ratio decreased in both genotypes over sessions, APPPS1-21 needed more CTs as they still respond more according to the previously learned rule **(D)**. Data plotted as mean ± SEM, *p < 0.05, **p < 0.01, ***p < 0.001 (Bonferroni *post hoc* test, APPPS1-21 vs. wild type controls).

As shown in Figures 4, 5, a similar behavioral profile is observed at 9 months. During VD, main effects of session are found in accuracy, number of CTs and Ct/RT ratio (*F*_(9,333)_ = 5.42, *p* < 0.001; *F*_(9,333)_ = 4.96, *p* < 0.001 and *F*_(9,333)_ = 3.39, *p* < 0.001, respectively), indicating a general learning effect across sessions. No significant differences between genotypes were found. During reversal sessions, next to an evident learning curve for all parameters (accuracy: *F*_(9,324)_ = 41.36, *p* < 0.001; trials: *F*_(9,324)_ = 37.08, *p* < 0.001; CTs: *F*_(9,324)_ = 39.31, *p* < 0.001; ratio: *F*_(9,324)_ = 37.27, *p* < 0.001, Figure 5), we again find a reversal learning impairment in APPPS1-21 animals. Transgenic animals have a lower performance accuracy (*F*_(1,324)_ = 11.97, *p* = 0.001), perform less RTs (*F*_(1,324)_ = 12.63, *p* = 0.001) and have a higher CT/RT ratio (*F*_(1,324)_ = 8.16, *p* = 0.007). Only for the number of CTs performed, we do not find a main effect of genotype (*F*_(1,324)_ = 0.73, *p* = 0.398). We also find an interaction for accuracy between session and genotype (*F*_(9,324)_ = 2.57, *p* = 0.007), RTs (*F*_(9,324)_ = 3.55, *p* < 0.001) and CTs (*F*_(9,324)_ = 3.02, *p* = 0.002). Figure 5 again shows that both genotypes set off performing similarly, but APPPS1-21 animals start lagging behind after 3–4 sessions.

## Discussion

We tested discrimination and reversal learning in 6- and 9-month-old APPPS1-21 mice using touchscreen operant cages. In AD patients, cognitive flexibility is affected at an early disease stage (Amieva et al., 2003; Ready et al., 2003; McGuinness et al., 2009). We, therefore, expected our transgenic animals to show reduced performance during the reversal phase that requires cognitive flexibility. We indeed found that the APPPS1-21 model displays robust touchscreen reversal learning deficits, at a stage of pathology that was previously considered to be early and non-symptomatic (Radde et al., 2006; Serneels et al., 2009; Lo et al., 2013). At both 6 and 9 months, transgenic animals need more sessions to reach criterion performance compared to wild-type controls. During VD, performance between both genotypes does not differ, indicating the observed difference cannot be attributed to impaired VD learning as such, but in cognitive flexibility.

When performing a more detailed analysis of the reversal phase, the number of RTs per session was significantly lower in the transgenic animals, whereas the number of CTs is similar or higher in APPPS1-21 mice. This indicates these animals are not less motivated or exhibit motor deficits, but need more CTs to adapt to the new reward contingency. Therefore, we calculated the CT/RT ratio, which denotes the persistence in the previously learned reward rule. This measure is significantly higher during REV trials in both 6- and 9-month-old APPPS1-21 mice, which indicates these animals have greater difficulty adapting to changing situations. When looking at accuracy, measured by percentage correct, we also find that the transgenic animals perform significantly worse from controls. Furthermore, we find a significant interaction between session and genotype, indicating that while the initial performance might be similar between genotypes, APPPS1-21 soon start lagging behind. When looking at Figures 3B, 5B, we can see the learning curves of both transgenic and control mice evolve similarly during the first sessions, but after reaching at and near 50 percent correct, the curves of the APPPS1-21 mice start to level off. According to Chudasama and Robbins (2003), performing below chance-level points at perseverative behavior, meaning the animals are not able to come off the formerly learned reward rule. Chance-level performance, on the other hand, indicates they are not able to learn the new reward rule. APPPS1-21 animals seem to be able to abandon a previously learned rule but have difficulty adopting a new one with changed reward contingencies.

When considering the course of the experiment, we opted for an individual criterion based protocol (instead of a fixed training period or group criterion, Mar et al., 2013), meaning that each animal advances to the next phase when it meets the performance criterion of the current phase. The advantage is, that all animals perform equally well when moving to the next stage (i.e., no over-training or failing to acquire initial reward rule). However, some animals might never reach criterion (and are excluded from analyses) and individual trajectories might differ markedly. As a consequence, a problem of missing values and loss of power arises inevitably in the course of sessions. Therefore, we decided to include dummy values (as explained in the “Statistical Analysis” paragraph). When comparing graphs with and without dummy values, barely any differences were detected, assuring this adjustment did not affect the statistical results.

Standard setups to test reversal learning, such as mazes and operant chambers, have some disadvantages. Performance in a water maze can be influenced by stress, motor impairments or visual problems. Dry mazes potentially induce unwanted odor cues and often depend on exploratory activity and motor functioning (Bussey et al., 2008), while fear conditioning often requires aversive stimuli such as electric shocks. In addition, all of these protocols show high variability between laboratories, partially due to differences in protocol, procedure or experimental apparatus used (Crabbe et al., 1999; Tecott and Nestler, 2004). Furthermore, despite efforts to standardize these tests, environmental and experimenter differences (way of handling the animals, odor cues, etc.) continue to induce unwanted variability that may obscure the subtle effects of genetic or other manipulations (Crabbe et al., 1999; Wahlsten et al., 2003; Bailey et al., 2006; Lewejohann et al., 2006; Mandillo et al., 2008). The present methodology and the use of touchscreen operant cages may help to eliminate or reduce interference and bias (Bussey et al., 2008, 2012; Mar et al., 2013; Horner et al., 2013). The protocol is low-stress and non-aversive, which eliminates potential effects of stressful and aversive manipulations (Joëls and Baram, 2009), and it does not depend on motor or olfactory proficiency. Automatization and standardization of the procedure minimizes variability and confounds by reducing within- and between-trial experimenter bias.

In addition to all of the technical advantages mentioned above, our mouse protocol is very similar to testing procedures used in clinical setting. Notably, a recent study, using an identical touchscreen test in mice and human subjects that carried mutations in a homologous gene, found similar results in both species (Nithianantharajah et al., 2015). Translatability of testing procedures and experimental variables means tremendous added value, especially in the field of AD research, where predictive and construct validity of preclinical research methods have been of much concern (Shepherd et al., 2016). Few studies have used such translatable methodology to measure cognitive functioning in mouse AD models. Romberg et al. (2013) found sustained attention, behavioral flexibility and memory defects in 4–5-month-old TgCRND8 mice with severe amyloid pathology. Furthermore, it was shown that impaired touchscreen attention in 9-month-old 3xTgAD mice could be rescued by Donepezil (Romberg et al., 2011).

## Ethics Statement

All experiments were reviewed and approved in accordance with EC directives by the Ethische Commissie Dierproeven (animal ethics commission), University of Leuven (KU Leuven), Belgium.

## Author Contributions

LVB, ZC-V and RD’H planned the study and wrote the manuscript. LVB and PH performed the experiments and analyzed the data. LVB, PH and RD’H revised the manuscript.

## Conflict of Interest Statement

The authors declare that the research was conducted in the absence of any commercial or financial relationships that could be construed as a potential conflict of interest.

## References

[B1] Amieva H. Phillips L. H. Della Sala S. Henry J. D. (2003). Inhibitory functioning in Alzheimer’s disease. Brain 127, 949–964. 10.1093/brain/awh045 14645147

[B2] Auersperg A. M. I. Gajdon G. K. von Bayern A. M. P. (2012). A new approach to comparing problem solving, flexibility and innovation. Commun. Integr. Biol. 5, 140–145. 10.4161/cib.18787 22808317 PMC3376048

[B3] Bailey K. R. Rustay N. R. Crawley J. N. (2006). Behavioral phenotyping of transgenic and knockout mice: practical concerns and potential pitfalls. ILAR J. 47, 124–131. 10.1093/ilar.47.2.124 16547369

[B4] Binetti G. Magni E. Padovani A. Cappa S. F. Bianchetti A. Trabucchi M. (1996). Executive dysfunction in early Alzheimer’s disease. J. Neurol. Neurosurg. Psychiatry 60, 91–93. 10.1136/jnnp.60.1.91 8558161 PMC486198

[B5] Bussey T. J. Holmes A. Lyon L. Mar A. C. McAllister K. A. L. Nithianantharajah J.. (2012). New translational assays for preclinical modelling of cognition in schizophrenia: the touchscreen testing method for mice and rats. Neuropharmacology 62, 1191–1203. 10.1016/j.neuropharm.2011.04.011 21530550 PMC3168710

[B6] Bussey T. J. Padain T. L. Skillings E. Winters B. D. Morton J. Saksida L. M. (2008). The touchscreen cognitive testing method for rodents: how to get the best out of your rat. Learn. Mem. 15, 516–523. 10.1101/lm.987808 18612068 PMC2505319

[B7] Chudasama Y. (2011). Animal models of prefrontal-executive function. Behav. Neurosci. 125, 327–343. 10.1037/a0023766 21639603

[B8] Chudasama Y. Robbins T. W. (2003). Dissociable contributions of the orbitofrontal and infralimbic cortex to pavlovian autoshaping and discrimination reversal learning: further evidence for the functional heterogeneity of the rodent frontal cortex. J. Neurosci. 23, 8771–8780. 10.1523/JNEUROSCI.23-25-08771.2003 14507977 PMC6740430

[B9] Crabbe J. C. Wahlsten D. Dudek B. C. Sibilia M. Wagner E. F. (1999). Genetics of mouse behavior: interactions with laboratory environment. Science 284, 1670–1672. 10.1126/science.284.5420.1670 10356397

[B10] Crowell T. A. Luis C. A. Vanderploeg R. D. Schinka J. A. Mullan M. (2010). Memory patterns and executive functioning in mild cognitive impairment and Alzheimer’s disease. Aging Neuropsychol. Cogn. 9, 288–297. 10.1076/anec.9.4.288.8772

[B11] Cummings J. L. Morstorf T. Zhong K. (2014). Alzheimer’s disease drug-development pipeline: few candidates, frequent failures. Alzheimers Res. Ther. 6:37. 10.1186/alzrt269 25024750 PMC4095696

[B12] Emery N. J. Clayton N. S. (2004). The mentality of crows: convergent evolution of intelligence in corvids and apes. Science 306, 1903–1907. 10.1126/science.1098410 15591194

[B13] Hay M. Thomas D. W. Craighead J. L. Economides C. Rosenthal J. (2014). Clinical development success rates for investigational drugs. Nat. Biotechnol. 32, 40–51. 10.1038/nbt.2786 24406927

[B14] Horner A. E. Heath C. Hvoslef-Eide M. Kent B. A. Kim C. H. Nilsson S. R. O.. (2013). The touchscreen operant platform for testing learning and memory in rats and mice. Nat. Protoc. 8, 1961–1984. 10.1038/nprot.2013.122 24051959 PMC3914026

[B15] Joëls M. Baram T. Z. (2009). The neuro-symphony of stress. Nat. Rev. 10, 459–466. 10.1038/nrn2632 19339973 PMC2844123

[B16] Klanker M. Feenstra M. Denys D. (2013). Dopaminergic control of cognitive flexibility in humans and animals. Front. Neurosci. 7:201. 10.3389/fnins.2013.00201 24204329 PMC3817373

[B17] Lewejohann L. Reinhard C. Schrewe A. Brandewiede J. Haemisch A. Görtz N.. (2006). Environmental bias? Effects of housing conditions, laboratory environment and experimenter on behavioral tests. Genes Brain Behav. 5, 64–72. 10.1111/j.1601-183x.2005.00140.x 16436190

[B18] Lo A. C. Callaerts-Vegh Z. Nunes A. F. Rodrigues C. M. P. D’Hooge R. (2013). Tauroursodeoxycholic acid (TUDCA) supplementation prevents cognitive impairment and amyloid deposition in APP/PS1 mice. Neurobiol. Dis. 50, 21–29. 10.1016/j.nbd.2012.09.003 22974733

[B19] Mandillo S. Tucci V. Hölter S. M. Meziane H. Banchaabouchi M. A. Kallnik M.. (2008). Reliability, robustness, and reproducibility in mouse behavioral phenotyping: a cross-laboratory study. Physiol. Genomics 34, 243–255. 10.1152/physiolgenomics.90207.2008 18505770 PMC2519962

[B20] Mar A. C. Horner A. E. Nilsson S. R. O. Alsiö J. Kent B. Kim C. H.. (2013). The touchscreen operant platform for assessing executive function in rats and mice. Nat. Protoc. 8, 1985–2005. 10.1038/nprot.2013.123 24051960 PMC4131754

[B21] McGonigle P. Ruggeri B. (2014). Animal models of human disease: challenges in enabling translation. Biochem. Pharmacol. 87, 162–171. 10.1016/j.bcp.2013.08.006 23954708

[B22] McGuinness B. Barret S. L. Craig D. Lawson J. Passmore A. P. (2009). Executive functioning in Alzheimer’s disease and vascular dementia. Int. J. Geriatr. Psychiatry 25, 562–568. 10.1002/gps.2375 19810010

[B23] Nithianantharajah J. McKechanie A. G. Stewart T. J. Johnstone M. Blackwood D. H. St Clair D.. (2015). Bridging the translational divide: identical cognitive touchscreen testing in mice and humans carrying mutations in a disease-relevant homologous gene. Sci. Rep. 5:14613. 10.1038/srep14613 26423861 PMC4589696

[B24] Radde R. Bolmont T. Kaeser S. A. Coomaraswamy J. Lindau D. Stoltze L.. (2006). Aβ-driven cerebral amyloidosis in transgenic mice reveals early and robust pathology. EMBO Rep. 7, 940–946. 10.1038/sj.embor.7400784 16906128 PMC1559665

[B25] Ready R. E. Ott B. R. Grace J. Cahn-Weiner D. A. (2003). Apathy and executive dysfunction in mild cognitive impairment and Alzheimer disease. Am. J. Geriatr. Psychiatry 11, 222–228. 10.1176/appi.ajgp.11.2.222 12611752

[B26] Reiman E. M. (2017). Putting AD treatments and biomarkers to the test. Nat. Rev. Neurol. 13, 74–76. 10.1038/nrneurol.2017.1 28084326

[B27] Romberg C. Horner A. E. Bussey T. J. Saksida L. M. (2013). A touch screen-automated cognitive test battery reveals impaired attention, memory abnormalities, and increased response inhibition in the TgCRND8 mouse model of Alzheimer’s disease. Neurobiol. Aging 34, 731–744. 10.1016/j.neurobiolaging.2012.08.006 22959727 PMC3532594

[B28] Romberg C. Mattson M. P. Mughal M. R. Bussey T. J. Saksida L. M. (2011). Impaired attention in the 3xTgAD mouse model of Alzheimer’s disease: rescue by donepezil (Aricept). J. Neurosci. 31, 3500–3507. 10.1523/JNEUROSCI.5242-10.2011 21368062 PMC3066152

[B29] Roth G. Dicke U. (2005). Evolution of the brain and intelligence. Trends Cogn. Sci. 9, 250–257. 10.1016/j.tics.2005.03.005 15866152

[B30] Serneels L. Van Biervliet J. Craessaerts K. Dejaegere T. Horré K. Van Houtvin T.. (2009). γ-Secretase heterogeneity in the Aph1 subunit: relevance for Alzheimer’s disease. Science 324, 639–642. 10.1126/science.1171176 19299585 PMC2740474

[B31] Shepherd A. Tyebji S. Hannan A. J. Burrows E. L. (2016). Translational assays for assessment of cognition in rodent models of Alzheimer’s disease and dementia. J. Mol. Neurosci. 60, 371–382. 10.1007/s12031-016-0837-1 27637601

[B32] Tabert M. H. Manly J. J. Liu X. Pelton G. H. Rosenblum S. Jacobs M.. (2006). Neuropsychological conversion to Alzheimer disease in patients with mild cognitive impairment. Arch. Gen. Psychiatry 63, 916–924. 10.1001/archpsyc.63.8.916 16894068

[B33] Tecott L. H. Nestler E. J. (2004). Neurobehavioral assessment in the information age. Nat. Neurosci. 7, 462–466. 10.1038/nn1225 15114359

[B34] Tranter L. J. Koutstaal W. (2008). Age and flexible thinking: and experimental demonstration of the beneficial effects of increased cognitively stimulating activity on fluid intelligence in healthy older adults. Neuropsychol. Dev. Cogn. 15, 184–207. 10.1080/13825580701322163 17851980

[B35] Van Harten A. C. Smits L. L. Teunissen C. E. Visser P. J. Koene T. Blankenstein M. A.. (2013). Preclinical AD predicts decline in memory and executive functions in subjective complaints. Neurology 81, 1409–1416. 10.1212/wnl.0b013e3182a8418b 24049134

[B36] van Schaik C. P. Burkart J. M. (2011). Social learning and evolution: the cultural intelligence hypothesis. Philos. Trans. R. Soc. Lond. B Biol. Sci. 366, 1008–1016. 10.1098/rstb.2010.0304 21357223 PMC3049085

[B37] Wahlsten D. Metten P. Phillips T. J. Boehm S. L. II. Burkhart-Kasch S. Dorow J.. (2003). Different data from different labs: lessons from studies of gene-environment interaction. J. Neurobiol. 54, 283–311. 10.1002/neu.10173 12486710

[B38] Windisch M. (2014). Mouse models of Alzheimer’s disease: promise, problems, and premises. Alzheimers Dement. 10:P478 10.1080/23262133.2017.1327002

